# A compressed sensing-based iterative algorithm for CT reconstruction and its possible application to phase contrast imaging

**DOI:** 10.1186/1475-925X-10-73

**Published:** 2011-08-18

**Authors:** Xueli Li, Shuqian Luo

**Affiliations:** 1 College of Biomedical Engineering, Capital Medical University, Beijing 100069, China

## Abstract

**Background:**

Computed Tomography (CT) is a technology that obtains the tomogram of the observed objects. In real-world applications, especially the biomedical applications, lower radiation dose have been constantly pursued. To shorten scanning time and reduce radiation dose, one can decrease X-ray exposure time at each projection view or decrease the number of projections. Until quite recently, the traditional filtered back projection (FBP) method has been commonly exploited in CT image reconstruction. Applying the FBP method requires using a large amount of projection data. Especially when the exposure speed is limited by the mechanical characteristic of the imaging facilities, using FBP method may prolong scanning time and cumulate with a high dose of radiation consequently damaging the biological specimens.

**Methods:**

In this paper, we present a compressed sensing-based (CS-based) iterative algorithm for CT reconstruction. The algorithm minimizes the *l_1_-*norm of the sparse image as the constraint factor for the iteration procedure. With this method, we can reconstruct images from substantially reduced projection data and reduce the impact of artifacts introduced into the CT reconstructed image by insufficient projection information.

**Results:**

To validate and evaluate the performance of this CS-base iterative algorithm, we carried out quantitative evaluation studies in imaging of both software Shepp-Logan phantom and real polystyrene sample. The former is completely absorption based and the later is imaged in phase contrast. The results show that the CS-based iterative algorithm can yield images with quality comparable to that obtained with existing FBP and traditional algebraic reconstruction technique (ART) algorithms.

**Discussion:**

Compared with the common reconstruction from 180 projection images, this algorithm completes CT reconstruction from only 60 projection images, cuts the scan time, and maintains the acceptable quality of the reconstructed images.

## Background

Computed Tomography (CT), which obtains a series of projection data of objects concerned from several view angles, can get the tomograms of the objects through the technology of image reconstruction. From a purely mathematical standpoint, the solution to the problem of how to reconstruct a function from its projections dated back to the paper by Radon in 1917. The current excitement in tomographic imaging originated with Hounsfield's invention of the X-ray computed tomographic scanner for which he received a Nobel Prize in 1972 [1]. The algorithms of image reconstruction from projections can be divided into two classes: the analytical method and the algebraic method [2]. The advantages of the analytical method, such as filtered back projection (FBP) method, are relatively high computational speed and short computational time. When the projection data are densely sampled, images can be reconstructed accurately with analytic methods [3]. Thus, this method is widely used in the commercial CT systems. However, if data containing a reduced number of projections sparsely sampled over an angular range are considered, the analytic algorithms will yield reconstructed images with severe aliasing artifacts such as sharp streaks [4]. The iterative algorithms, on the contrary, can reconstruct images from relatively less projection data. But, it will take much longer time with iterative algorithms versus analytic algorithms.

In real-world applications, especially the biomedical applications, higher temporal resolution and lower radiation dose have been constantly pursued. One can reduce scanning time and radiation dose by decreasing X-ray exposure time at each projection view. However, the reduction of exposure time would further lower the signal-to-noise ratio (SNR) of the projection images and consequently lower the reconstructed images' quality [5]. The other approach to decrease scanning time and radiation dose is to reduce the number of projections.

Compressed sensing theory, also known as compressive sampling or CS, suggested by Candès, Romberg, Tao and Donoho in 2006 [6,7], states that one can reconstruct images accurately from a number of samples that are far smaller than the desired resolution of the images [8]. Inspired by the theory's success in signal recovery, we have anticipated that a CS-based algorithm may be used to reconstruct images from substantially reduced projection data. The algorithm minimizes the *l_1_*-norm of the sparse image as the constraint factor for the iteration procedure. This work focuses on reconstructing images from significantly reduced projection data, shortening scanning time, minimizing radiation dose without reducing image quality, and employing this algorithm in phase contrast imaging experiments.

The paper is organized as follows. In section 2, the experimental set-up will be introduced for our polystyrene sample imaging. In section 3, the materials and methods for data scanning and image reconstruction will be described, in section 4, numerical results under different conditions are reported and the reconstructed and reference images at the visualization-based and quantitative-metric-based evaluation levels are compared. Finally, the implication of the results will be further discussed in section 5.

### The experimental set-up for phase contrast imaging

Phase contrast X-ray imaging [9-13] enables the observation of light samples, such as biological soft tissue, without a contrast agent, because the phase shift cross sections of light elements are much larger than their absorption cross sections [14]. However, in the research of phase contrast imaging, the FBP method has been commonly exploited in CT reconstruction [15-17]. Applying the FBP method requires a large amount of projection data, which can prolong scanning time and cumulate with a high dose of radiation potentially damaging the biological specimens.

**Figure 1:**
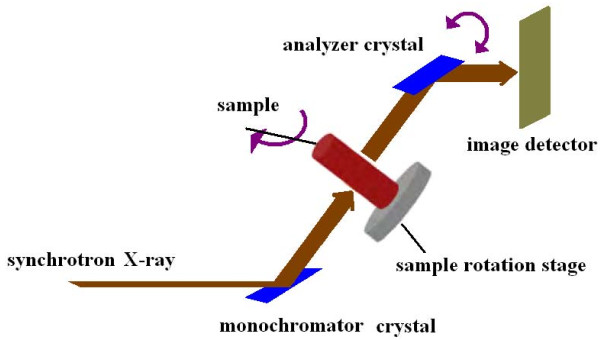
**Schematic set-up of an analyzer based X-ray imaging system**.

Our experiment was performed at the 4W1A beamline of Beijing Synchrotron Radiation Facilities (BSRF). The synchrotron X-ray beam was 20 mm in width by 10 mm in height. The X-ray beam energy was set at 15keV in this experiment. The distance between the synchrotron radiation source and the sample was approximately 43 m. The X-ray CCD was the Photonic Science X-ray FDI-18 mm camera system with 1300 × 1030 pixels, and 10.9 × 10.9 μm^2 ^per pixel. The schematic set-up of this analyzer based imaging (ABI) system is shown in Figure 1. It composes of a monochromator crystal, an analyzer crystal, one sample rotation stage, and one image detector. The monochromator crystal is used to produce highly parallel and monochromatic X-ray beams. When the highly parallel and monochromatic X-ray beams travel through the object, their directions change due to refraction and scattering. According to the Brag diffraction theory, the analyzer crystal only reflects photons coming from a particular angle. Thus, if the analyzer crystal is rotated about an axis perpendicular to the meridian plane, the diffracted intensity will trace out a 'rocking curve' [18]. We obtain the projection data of our polystyrene phantom when the analyzer crystal was set to the half intensity points on the high-angle sides of the rocking curve.

## Materials and methods

**Figure 2:**
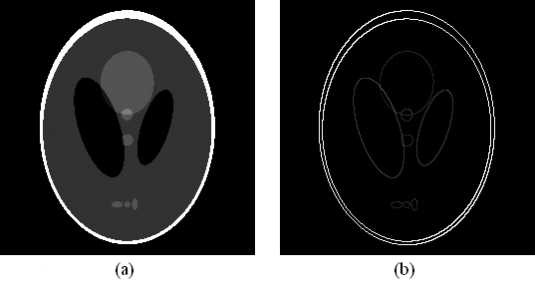
**Shepp-Logan phantom**. (a) the original image, (b) the gradient counterpart of (a).

**Figure 3:**
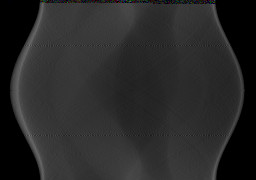
**Projection data of Shepp-Logan phantom in the 2D data space with the horizontal and vertical axes representing the detector-bin and view-angle coordinates, respectively**.

**Figure 4:**
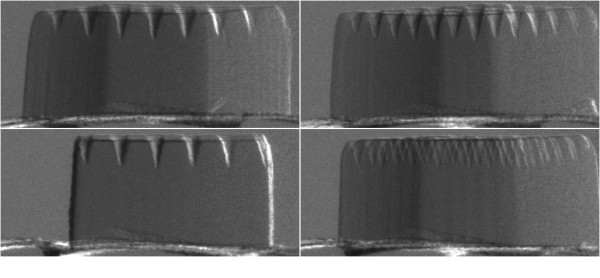
**The Radiographs collected from the Diffraction enhanced X-ray imaging system in different rotate angles**.

Both software phantom and real sample were used to test our algorithm. The software phantom was a discrete 256 × 256 Shepp-Logan phantom (see Figure 2(a)). We generated Sheep-Logan phantom using Matlab command sentence "phantom(256)". We suppose that it is the desired CT image and each pixel value presents an attenuation coefficient. To generate the projection data, we simulated the procedure of an X-ray scan, computed line integrals across the image, and eventually, obtained the projection data as shown in Figure 3. More popularly, this projection data is named 'sinogram' [19]. The horizontal and vertical axes represent the detector-bin and view-angle coordinates. In our sample, the number of the detector-bin was 256, and the number of the view-angle had two selections which were 60 and 30. Since minimal levels of noise were introduced to assess the stability of the algorithm, such studies were supposed to give an upper bound to the performance of the CT reconstruction algorithms. The real sample was a polystyrene hexahedron. The length, width, and height of this hexahedron were about 4, 4, and 2 mm respectively. On one 4 mm by 4 mm surface, several lines of holes were punched by a laser gun. We placed the other 4 mm by 4 mm surface on the sample stage. By scanning the sample over 180° with the angular step of 1°, we obtained one hundred and eighty projection images of 1300 × 1030 pixels. Several pieces of projection images in different angles of rotation are shown in Figure 4. Before reconstruction, we moved the background of the projection images. And since the sample was not placed at the absolute center of the sample rotation stage, the projection images should be cut to a suitable size to compose 'sinogram'.

Now, we describe our CS-based iterative algorithm for image reconstruction in parallel-beam CT. A successful application of CS requires that the desired image should have a sparse representation in a known transform domain [7]. Consider an image ***f***, which can be viewed as an *N × 1 *column vector in *R^N^*, whose individual elements *f_j_, j *= 1, 2, ... N are *N *pixel values of the image. Expand vector ***f ***in an orthonormal basis *Ψ *as follows:

(1) f=Ψx,

where *Ψ *is the *N *by *N *matrix [*ψ*_1_..., *ψ_N_*] with the *N × 1 *vectors ${\left\{\psi_{i}\right\}}_{i=1}^{N}$ as columns and ***x ***is also an *N × 1 *column vector. If all but a few of entries in vector ***x ***are zero or almost zero, we will say that ***f ***is sparse in the *Ψ *domain and ***x ***is its sparse representation. For example, the Shepp-Logan phantom in Figure 2(a) and its gradient counterpart in 2(b), we denote the intensity of pixel of a 2D image as *f_h, w_*, where *h = 1, 2... H; w = 1, 2... W*; *H *and *W *are the height and width of the 2D image respectively and *W × H = N*. If the pixel values are labeled by *f_h, w_*, the gradient modulus is as follows.

(2) $$\left|\nabla f_{h,w}\right|=\sqrt{{\left(f_{h+1,w}-f_{h,w}\right)}^{2}+{\left(f_{h,w+1}-f_{h,w}\right)}^{2}}.$$

We refer to this quantity as the gradient image [20]. The number of non-zero pixels in this *256 × 256 *image (Figure 2(a)) is 27521, while the number of non-zero pixels in its gradient image (Figure 2(b)) is only 2182, which is much less than the pixel number of the image. That means clearly, ***f ***and ***x ***are equivalent representations of the image, with ***f ***in the space domain and ***x ***in the *Ψ *domain. In realistic CT imaging, suppose the sampled parallel-beam projection-data of image *f *are modeled by a discrete linear system

(3) g=Φf,

where vector ***g ***has length *M *with individual measurements referred to as *g_i_, i *= 1, 2, ... M and *Φ *is the *M *by *N *system matrix [21] that yields the discrete set of projection measurements for parallel-beam scanning from the object. Then substituting ***Ψx ***for ***f ***, ***g ***can be written as

(4) $$g=\Phi f=\Phi \Psi x=\Phi^{\prime }x,$$

where Φ*' = *Φψ is an *M *by *N *matrix. For a sparse image, since *M < N *in Eq. (4) there are infinitely many $\tilde{x}$ that satisfy $g=\Phi \prime \tilde{x}$. Therefore, the image reconstruction is aimed at finding the vector ***x ***in transform domain by solving the linear program [6-8]

(5) $$x=\underset{\tilde{x}}{\mathrm{argmin}}{∥\tilde{x}∥}_{l1}\text{subject to}\;\Phi^{\prime }\tilde{x}=g.$$

Define the *l_1 _-*norm of the vector ***x ***as ${∥x∥}_{l_{1}}=\sum_{i=1}^{N}\left|x_{i}\right|$[8]. In this paper, specifically, ***x ***represents the gradient image vector of the desired image.

The constraints $\Phi^{\prime }\tilde{x}=g$ can be satisfied under the circumstance that measurements contain no noise. But it is unattainable, so we consider the constraints that

(6) $$\left|\Phi^{\prime }\tilde{x}-g\right|<\varepsilon ,$$

where ε is a small error factor.

To minimize the *l_1 _-*norm of the gradient image [20,22,23], a basic gradient descent method was employed. Usually, the gradient descent method is to reduce the objective function $\chi^{2}={∥\text{g}-\Phi f∥}^{2}$ by iteratively moving the image along the gradient [24]

(7) $$f^{next}=f^{current}-\alpha {\vec{\Delta }}^{current},$$

where *α *is constant to control the descent speed and $\vec{\Delta }$ is related to the gradient of the *l_1 _-*norm of the gradient image which can also be thought of an image. Each pixel value of it is expressed as the following partial derivative [20]

(8) $$\begin{array}{c} v_{h,w}=\frac{\partial {∥\nabla f_{h,w}∥}_{l_{1}}}{\partial f_{h,w}}=\frac{2f_{h,w}-f_{h+1,w}-f_{h,w+1}}{\sqrt{\varepsilon +{\left(f_{h+1,w}-f_{h,w}\right)}^{2}+{\left(f_{h,w+1}-f_{h,w}\right)}^{2}}}\\ +\frac{f_{h,w}-f_{h-1,w}}{\sqrt{\varepsilon +{\left(f_{h,w}-f_{h-1,w}\right)}^{2}+{\left(f_{h-1,w+1}-f_{h-1,w}\right)}^{2}}}\\ +\frac{f_{h,w}-f_{h,w-1}}{\sqrt{\varepsilon +{\left(f_{h+1,w-1}-f_{h,w-1}\right)}^{2}+{\left(f_{h,w}-f_{h,w-1}\right)}^{2}}} \end{array}.$$

To avoid the condition that the denominator vanishes, a small positive number ε is added in each radical sign.

In the discrete setting, the parallel-beam projection-data vector $\vec{g}$ can be written as weighted sums over the pixels traversed by the X-ray as

(9) $$g_{i}=\overset{N}{\underset{j=1}{\sum }}\phi_{i,j}\cdot f_{j},\text{where}\;i=1,2,\cdots \;,M.$$

**Figure 5:**
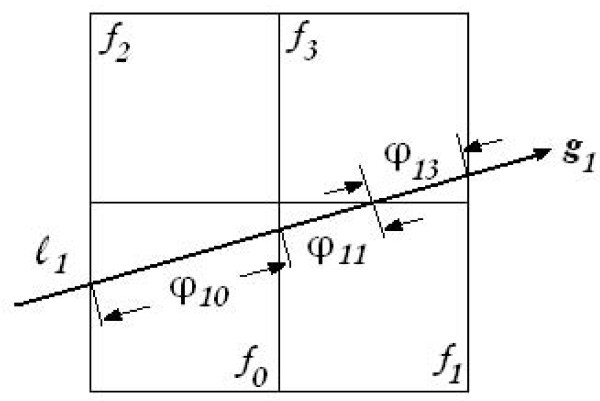
**Sensing matrix model**. *f_0_, f_1_, f_2_*, and *f_3 _*: four pixels. *φ_10_, φ_11_*, and *φ_13 _*: three weight coefficients. *l_1 _*is one X-ray, and *g_1 _*is one projection value.

**Figure 6:**
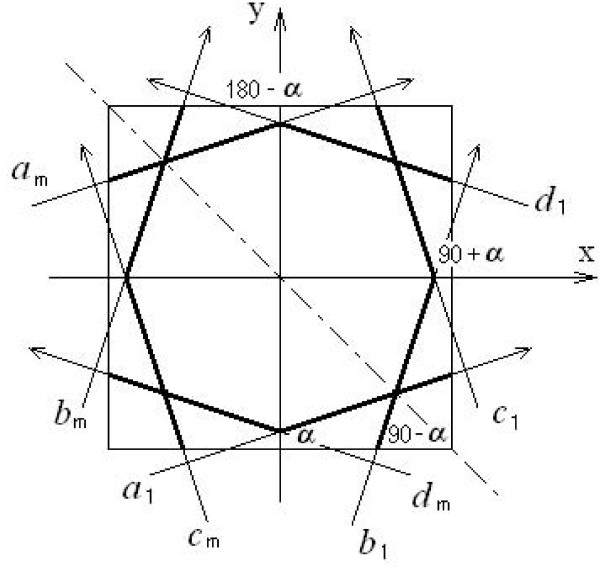
**Computation of sensing matrix weights model**. Eight arrow lines represent X-rays. The rays at different angles are labeled by different letters *a, b, c*, and *d*. The subscripts represent the detector array's position. α, (90- α), (90+ α) and (180- α): angles from X-axis to X-rays.

The weight component *φ_i, j _*of the system matrix Φ is computed by the intersection length of the *i*th ray through the *j*th pixel. Using a sketch we can understand it clearly. In Figure 5, the image is composed of four pixels *f*_0_, *f*_1_, *f*_2_, and *f*_3_; *g*_1 _is a measurement; and the X-ray *l*_1 _passed pixels *f*_0_, *f*_1_, and *f*_3_; the lengths passed these three pixels are *φ*_1, 0_, *φ*_1, 1 _and *φ*_1, 3 _respectively. The computation of the weight *φ *is complex. The immediate computation of each *φ *will prolong the reconstruction time. Especially with iteration times' increasing, the computation of weights will repeat again and again. A solution is to save the weights in a file in advance and read the weights from this file during the iteration. Since the X-rays are parallel-beam, we can take advantage of the symmetrical characteristic of X-rays. In Figure 6, the X-rays are denoted by *a, b, c*, and *d*. Actually, the measurements obtained by the integral along the X-rays *a_1_, b_1_, c_1 _*and *d_1 _*are detected by the same detector in different rotate angles which are α, (90- α), (90+ α) and (180- α) degrees respectively. The *a_1_, a_2_*,..., *a_m _*is a set of parallel beams. These eight X-rays (*a_1_, a_m_, b_1_, b_m_, c_1_, c_m _, d_1 _*and *d_m_*) in Figure 6 have such symmetrical characteristics that line *y = -x *is the symmetrical axis of *a_1 _*and *b_1_*; the x-axis is the symmetrical axis of *b_1 _*and *c_1_*, also *a_1 _*and *d_1_*; and the parallel beams *a_1 _*and *a_m _, b_1 _*and *b_m _, c_1 _*and *c_m _, d_1 _*and *d_m _*, are symmetrical to the origin. So, if we have a weight value in X-ray *a_1 _*(the wide segment in line *a_1_*), we can gain the other seven weight values (the wide segment in the other seven lines) using the symmetrical characteristics.

In the following, we give the steps of the reconstruction algorithm in the form of a pseudo-code and abbreviated notation.

(1) initialization of the image *f*:

$$f^{\left(0\right)}=0;$$

(2) iterative process:

$$f_{j}^{\left(k\right)}=f_{j}^{\left(k-1\right)}+\lambda \frac{g_{i}-\overset{N}{\underset{n=1}{\sum }}\phi_{i,n}\cdot f_{n}^{\left(k-1\right)}}{\overset{N}{\underset{i=1}{\sum }}\phi_{i,n}^{2}}\cdot \phi_{i,j};$$

where the relaxation parameter λ [25] is a positive real number to adjust the iterative, and *k *is from 1 to M. When *k = M *, a complete iteration period was finished. The next iteration will enforce the estimated image to the constraint $\left|\Phi^{\prime }\tilde{x}-g\right|<\varepsilon$ by the gradient descent iteration.

(3) initialization of the gradient descent image:

$${\hat{f}}^{\left(0\right)}=f^{\left(\text{M}\right)};$$

(4) gradient descent iteration:

${\hat{f}}^{\left(l\right)}={\hat{f}}^{\left(l-1\right)}-\alpha \cdot \vec{\Delta },$, and

$$\vec{\Delta }=\left|{\hat{f}}^{\left(0\right)}-f^{\left(0\right)}\right|\cdot \frac{v_{y,x}}{\left|v_{y,x}\right|}.$$

In this iteration, the end time we selected is 5.

(5) Initialize the next iterative step:

$$f^{\left(0\right)}={\hat{f}}^{\left(end\right)},$$

then we repeat step (2) - (5) until the difference between the current *f^(M) ^*and the previous *f^(M) ^*is smaller than the threshold we set or the iteration number is more than 1000.

About the control parameters, we selected *λ *= 1.0, *ε *= 0.0001, and *α *= 0.5 respectively. The threshold value to stop iteration was set as 0.001. These presetting parameters and coefficients only appear to alter the convergence rate.

## Results

To demonstrate this CS-based iterative algorithm for image reconstruction from under-sampled projection data, we performed two sets of studies: the first set of studies were designed in such a way as to acquire some theoretical understanding of how the CS-based iterative algorithm performs on image reconstruction from reduced projection data with the parallel-beam configuration under ideal conditions, and the second set of numerical examples aimed to see how the CS-based iterative algorithm could be applied to phase contrast CT image reconstruction.

Recall Eq. 3, in the situation that the measurement data *g *contain no noise and the full scan views data are used, one might expect to reconstruct images accurately. However, in the present studies, the projection data were under-sampled in view angle. We performed the FBP, traditional algebraic reconstruction technique (ART) and CS-based iterative reconstruction algorithms under the condition that the numbers of views were 60 and 30. The image-quality evaluation for each specimen was performed at two different levels, including 1) visualization-based evaluation, and 2) quantitative-metric-based evaluation. Some of the evaluation concerns make a comparison between the reconstructed and original images.

**Figure 7:**
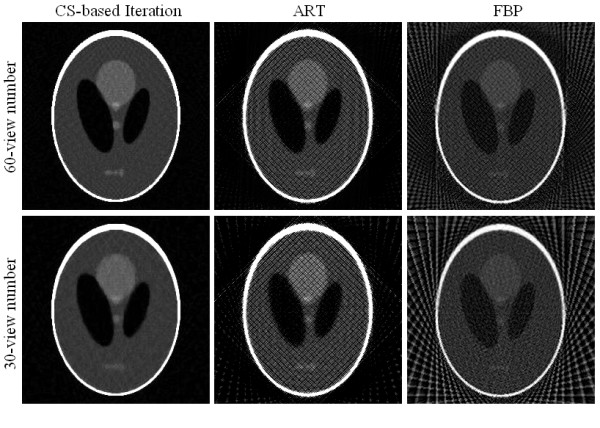
**Shepp-Logan phantom images reconstructed from 60 and 30 view numbers using CS-based iterative algorithm (column 1), ART (column 2), and FBP (column 3)**.

Visual inspection of reconstructions in Figure 7 suggests that under the conditions of few-view (60- and 30-view number) projection data, the CS-based iterative algorithm can effectively suppress streak artifacts and noise observed in images obtained with the FBP and traditional ART algorithms, thus yielding images with a higher visual similarity to the Shepp-Logan phantom image (see Figure 2(a)) than those obtained with other algorithms.

In addition to visualization-based evaluation, the following three metrics were employed to quantitatively assess the similarity between reconstructed images and the original phantom image: 1) the root mean squared error (RMSE), 2) the universal quality index (UQI) [26], and the correlation coefficient (CC), which are defined as

(10) $$RMSE=\sqrt{\frac{\overset{N}{\underset{i=1}{\sum }}{\left(f_{ri}-f_{0i}\right)}^{2}}{N}},$$

(11) $$UQI=\frac{2Cov\left\{f_{r},f_{0}\right\}}{D\left(f_{r}\right)+D\left(f_{0}\right)}\frac{2{\bar{f}}_{r}\cdot {\bar{f}}_{0}}{{{\bar{f}}_{r}}^{2}+{{\bar{f}}_{0}}^{2}},$$

(12) $$CC=\frac{2Cov\left\{f_{r},f_{0}\right\}}{\sqrt{D\left(f_{r}\right)}\cdot \sqrt{D\left(f_{0}\right)}},$$

where vector *f_r _*and *f_0 _*denote the reconstructed and original images of N pixels, and

$$\begin{array}{c} \begin{array}{cc} {\bar{f}}_{0}=\frac{1}{N}\overset{N}{\underset{i=1}{\sum }}f_{0i}, & {\bar{f}}_{r}=\frac{1}{N}\overset{N}{\underset{i=1}{\sum }}f_{ri},\\ D\left(f_{0}\right)=\frac{1}{N-1}\overset{N}{\underset{i=1}{\sum }}{\left(f_{0i}-{\bar{f}}_{0}\right)}^{2}, & D\left(f_{r}\right)=\frac{1}{N-1}\overset{N}{\underset{i=1}{\sum }}{\left(f_{ri}-{\bar{f}}_{r}\right)}^{2}, \end{array}\\ Cov\left\{f_{r},f_{0}\right\}=\frac{1}{N-1}\overset{N}{\underset{i=1}{\sum }}\left(f_{ri}-{\bar{f}}_{r}\right)\left(f_{0i}-{\bar{f}}_{0}\right). \end{array}$$

The RMSE is widely used for measuring reconstruction accuracy, whereas the UQI and CC can be used for evaluating the pixel-to-pixel similarity between reconstructed and original images. When assessing the image's quality, we demand the RMSE index to be as small as possible, while expecting the UQI and CC to have the contrary results.

In Figure 7, the images in the left column are the reconstructed images using CS-based iterative algorithm, the middle column using ART, and the right column using FBP. The images in row 1 are reconstructed from 60-view data, and in row 2 the images are reconstructed from 30-view data.

From the digital Shepp-Logan phantom and reconstructed images, we computed their RMSEs, UQIs, and CCs and summarized them in Table 1, 2, and 3 respectively. Results of these three metrics suggest that the CS-based iterative algorithm yields images more similar to the original image than the FBP and traditional ART algorithms.

**Table 1:** Quantitative assessment RMSE of Shepp-Logan phantom images

RMSE	CS-based Iteration	ART	FBP
60-view number	16.82	22.61	37.92
30-view number	17.26	25.07	40.76

**Table 2:** Quantitative assessment UQI of Shepp-Logan phantom images

UQI	CS-based Iteration	ART	FBP
60-view number	0.942	0.894	0.570
30-view number	0.938	0.822	0.490

**Table 3:** Quantitative assessment CC of Shepp-Logan phantom images

CC	CS-based Iteration	ART	FBP
60-view number	0.947	0.900	0.719
30-view number	0.945	0.891	0.646

**Figure 8:**
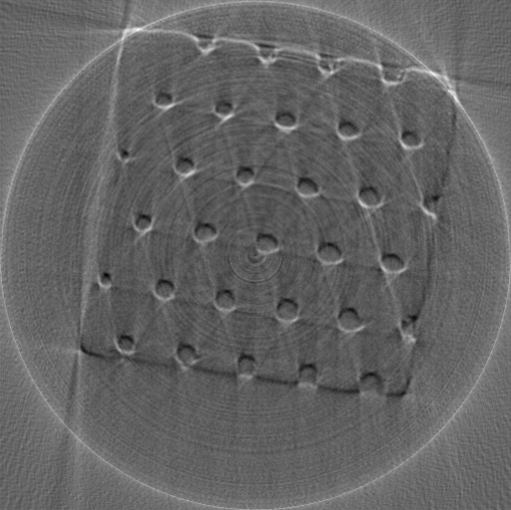
**The reference Polystyrene image reconstructed from 180-view projection data using FBP algorithm**.

**Figure 9:**
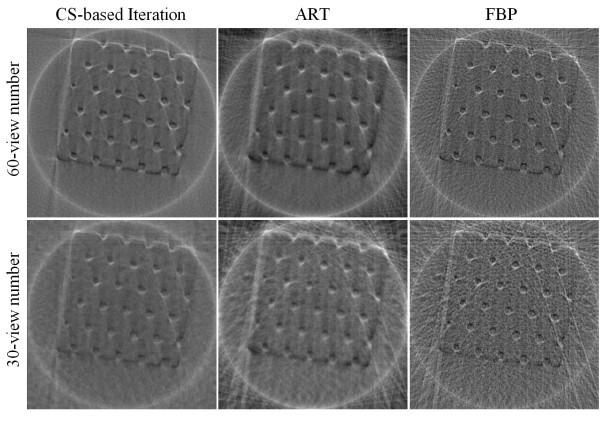
**Polystyrene phantom images reconstructed from 60 and 30 view numbers using CS-based iterative algorithm (column 1), ART (column 2), and FBP (column 3)**.

In addition to the simulated experiments with Shepp-Logan phantom, phase contrast X-ray imaging of a real polystyrene phantom was also performed. We collected 180 radiographs at 180 views. From this full data set, we applied the conventional FBP algorithm to yield the reference CT image in the 44th slice (see Figure 8). Then we extracted the 60- and 30-view subsets of data evenly distributed over π-view to simulate data collected at a reduced number of projection views. The reconstructed images are shown in Figure 9, and the three quantitative metrics RMSEs, UQIs, and CCs are computed and summarized in Table 4, 5, and 6 respectively. The results suggest that the CS-based iterative algorithm can yield the most similar images to the reference image. Different from the results of the reconstructed Shepp-Logan images, images reconstructed from the traditional ART algorithm seem to have the least similarity to the reference image. The reason might be because the reference image is reconstructed from the FBP algorithm as well.

**Table 4:** Quantitative assessment RMSE of **Polystyrene **phantom images

RMSE	CS-based Iteration	ART	FBP
60-view number	7.47	17.90	12.16
30-view number	9.52	19.44	17.37

**Table 5:** Quantitative assessment UQI of **Polystyrene **phantom images

UQI	CS-based Iteration	ART	FBP
60-view number	0.897	0.648	0.797
30-view number	0.817	0.584	0.613

**Table 6:** Quantitative assessment CC of **Polystyrene **phantom images

CC	CS-based Iteration	ART	FBP
60-view number	0.900	0.668	0.803
30-view number	0.831	0.601	0.635

## Discussion and Conclusions

In this article, a CS-base iterative algorithm reconstructing images from substantially reduced projection data was presented. Both the digital Shepp-Logan phantom and the real polystyrene sample experiment results show that the CS-based iterative algorithm can yield images with quality comparable to that obtained with existing FBP and traditional ART algorithms. However, when the number of gradient descent iterations is increased, the smoothing artifact in the reconstructed images will be more obvious. To improve this situation, reducing the number of gradient descent iterations or the step size is an alternative, but if the gradient descent is too small, the algorithm will reduce to the traditional ART algorithm.

Because the system matrix for sampling is saved in a matrix file, we must create a correlating matrix file for different images in different dimensions at first. The size of such a file, however, relates to the projection data's number and images' dimensions. For this reason, if either of these two elements is rather big, the matrix file will be quite large. Therefore, either compressing the matrix file further or diminishing the region of interest (ROI) in the reconstructed images may be one direction of our further work.

In conclusion, the paper aims to reduce the impact of artifacts introduced into the CT reconstructed image by insufficient projection information. The feasibility of this method which enforces the gradient descent for constraints in traditional iterative algorithms has been demonstrated by both the simulated phantom and the real polystyrene sample experiments. The results show that the CS-based iterative algorithm can yield images with quality comparable to that obtained with existing FBP and traditional ART algorithms. Further research will be performed to improve algorithm efficiency. Moreover, applying this algorithm to a less "sparse" sample such as the real biological soft tissues of small animals and studying how effective the method would be is our future concern.

## Competing interests

The authors declare that they have no competing interests.

## Authors' contributions

XL worked on the algorithm design and implementation, and wrote the paper; SL contributed to discussion and suggestions throughout this topic, including the manuscript writing. All authors read and approved the final manuscript.
